# Quantum Correlation Based on Uhlmann Fidelity for Gaussian States

**DOI:** 10.3390/e21010006

**Published:** 2018-12-22

**Authors:** Liang Liu, Jinchuan Hou, Xiaofei Qi

**Affiliations:** 1 College of Mechanics, Taiyuan University of Technology, Taiyuan 030024, China; 2 College of Mathematics, Taiyuan University of Technology, Taiyuan 030024, China; 3 Department of Mathematics, Shanxi University, Taiyuan 030006, China; 4 Institute of Big Data Science and Industry, Shanxi University, Taiyuan 030006, China

**Keywords:** quantum correlations, Gaussian states, Uhlmann fidelity, Gaussian unitary operators

## Abstract

A quantum correlation $N_{F}^{G,A}$ for (n+m)-mode continuous-variable systems is introduced in terms of local Gaussian unitary operations performed on Subsystem A based on Uhlmann fidelity *F*. This quantity is a remedy for the local ancilla problem associated with the geometric measurement-induced correlations; is local Gaussian unitary invariant; is non-increasing under any Gaussian quantum channel performed on Subsystem B;and is an entanglement monotone when restricted to pure Gaussian states in the (1+m)-mode case. A concrete formula for (1+1)-mode symmetric squeezed thermal states (SSTSs) is presented. We also compare $N_{F}^{G,A}$ with other quantum correlations in scale, such as Gaussian quantum discord and Gaussian geometric discord, for two-mode SSTSs, which reveals that $N_{F}^{G,A}$ has some advantage in detecting quantum correlations of Gaussian states.

## 1. Introduction

One of the main features of quantum mechanics in multipartite quantum systems is the presence of quantum correlation (QC). Though the entanglement is surely the most important among the QCs [1,2,3,4], the study and the characterization of QCs that go beyond the paradigm of entanglement have recently attracted more and more attention since non-entangled quantum correlations also play important roles in various quantum computing tasks and quantum communications [5,6,7].

Quantifying QCs for continuous-variable systems was carried out in various ways. G.Adesso and A. Datta [8] and P. Giorda and M. G. A. Paris [9] independently proposed Gaussian quantum discord (GQD). In [8], the authors analytically calculated the GQD for two-mode Gaussian states and claimed that almost all two-mode Gaussian states have quantum correlations. In [9], for squeezed thermal states (STSs), an entanglement threshold in terms of GQD was given. It is in general difficult to compute GQD since it involves a minimization process over all possible local Gaussian positive operator-valued measurements (GPOVMs) in a bipartition. The authors in [10] studied the computational complexity of quantum discord (QD) for finite-dimensional systems and found it increasing exponentially with the dimension of the Hilbert space. Many efforts have been made to find simpler methods to quantify these correlations. For example, in [11], G. Adesso and D. Girolami gave Gaussian geometric discord (GGD) for Gaussian states and provided an explicit formula for two-mode STSs, and in addition, they discussed other approaches to quantify Gaussian quadrature correlations. In analogy with the GQD, in [12], the measurement-induced disturbance (MID) of Gaussian states was studied by constraining the optimization to all bi-local GPOVMs, and an explicit formula for some families of states was given. In [13], measurement-induced nonlocality (MIN) for Gaussian states was discussed, and analytic formulas for two-mode STSs, as well as mixed thermal states were provided. Gaussian discord of response ($GD_{R}^{x}$) for two-mode Gaussian states can be found in [14]. For other related results, see [15,16,17,18,19,20,21,22] and the references therein.

Despite some efforts, almost all known quantifications of various correlations for continuous-variable systems are difficult to evaluate and can only be calculated for (1+1)-mode Gaussian states or some special states. Thus, it is natural and important to find more reliable and useful quantifications for QCs.

The purpose of this paper is to propose a correlation $N_{F}^{G,A}$ for continuous-variable systems in terms of local Gaussian unitary operations based on Uhlmann fidelity. We show that $N_{F}^{G,A}$ is a quantum correlation without the ancilla problem, is local Gaussian unitary invariant, is contained in any non-product state, is monotonically non-increasing under Gaussian quantum channels acting on the Subsystem B, and reduces to an entanglement measure for (1+m)-mode pure Gaussian states. Furthermore, we give a concrete formula for any two-mode symmetric squeezed thermal states (SSTSs) and compare $N_{F}^{G,A}$ with some other QCs.

Uhlmann fidelity was firstly proposed as a measure of closeness between two arbitrary states ρ and σ, defined as $F\left(\rho ,\sigma \right)={\left(\mathrm{Tr}\sqrt{\sqrt{\rho }\sigma \sqrt{\rho }}\right)}^{2}$ [23]. Uhlmann fidelity itself has many good properties, such as unitary invariance, monotonicity under quantum operations, and strong concavity [23,24,25]; and so it has many applications; for example, see [26,27,28,29,30,31] and the references therein. Recently, Uhlmann fidelity for Gaussian states was studied and some useful results were obtained in [32,33]. Though Uhlmann fidelity itself is not a metric, one can define a metric based on it as D(ρ,σ)=g(F(ρ,σ)), where *g* is a monotonically-decreasing function of *F*. Some well-known Uhlmann fidelity-induced metrics are the sine metric $C\left(\rho ,\sigma \right)=\sqrt{1-F(\rho ,\sigma )}$, Bures metric $B\left(\rho ,\sigma \right)={\left(2-2\sqrt{F(\rho ,\sigma )}\right)}^{\frac{1}{2}}$, and Bures angle $A\left(\rho ,\sigma \right)=\arccos\sqrt{F(\rho ,\sigma )}$ [34].

Although we may use any metric D(ρ,σ)=g(F(ρ,σ)) to introduce the quantum correlation by local unitary operations, in the present paper, we accept C(ρ,σ) as the metric for reasons of simplicity.

## 2. A Uhlmann Fidelity-Based Quantum Correlation and Its Properties

In this section, we define a QC $N_{F}^{G,A}$ by local unitary operations for (n+m)-mode states using the sine metric based on Uhlmann fidelity and discuss its properties.

We first recall some notions and notations.

For convenience, we denote by S(H) the set of all states of the quantum system described by the Hilbert space *H*. Assume that ρ∈S(H) is any state of an *n*-mode continuous-variable system with the state space $H=H_{1}⊗H_{2}⊗\cdots ⊗H_{n}$ ($\dim H_{k}=\infty$ for each k=1,2,…,n). The characteristic function of ρ is defined as $\chi_{\rho }\left(\alpha \right)=\mathrm{Tr}\left(\rho D\left(\alpha \right)\right)$, where $\alpha =\left(\alpha_{1},\alpha_{2},\cdots ,\alpha_{n}\right)\in C^{n}$, $D\left(\alpha \right)={⊗}_{k=1}^{n}D\left(\alpha_{k}\right)$ is the Weyl operator with $D\left(\alpha_{k}\right)=\exp\left(\alpha_{k}{\hat{a}}_{k}^{†}+\alpha_{k}^{*}{\hat{a}}_{k}\right)$ the Weyl operator of the $k^{\mathrm{th}}$ mode. Here, ${\hat{a}}_{k}=\left({\hat{Q}}_{k}+i{\hat{P}}_{k}\right)/\sqrt{2}$ and ${\hat{a}}_{k}^{†}=\left({\hat{Q}}_{k}-i{\hat{P}}_{k}\right)/\sqrt{2}$ are respectively the annihilation and creation operators in the $k^{\mathrm{th}}$ mode; $\hat{Q_{k}}$ and $\hat{P_{k}}$ respectively stand for the position and momentum operators satisfying the canonical commutation relations (CCR) $\left[{\hat{Q}}_{k},{\hat{P}}_{l}\right]=i\delta_{kl}I$ and $\left[{\hat{Q}}_{k},{\hat{Q}}_{l}\right]=\left[{\hat{P}}_{k},{\hat{P}}_{l}\right]=0$, k,l=1,2,⋯,n. ρ is called a Gaussian state if $\chi_{\rho }\left(\alpha \right)$ is of the form:

(1) $$\chi_{\rho }\left(\alpha \right)=\exp\left[-\frac{1}{4}\lambda_{\alpha }^{T}J\Gamma J\lambda_{\alpha }+iJd^{T}\lambda_{\alpha }\right],\;$$

where λα=2(Reα1,Imα1,⋯,Reαn,Imαn)∈R2n, $J={⊕}_{k=1}^{n}J_{k}={⊕}_{k=1}^{n}\left(\begin{array}{cc} 0 & 1\\ -1 & 0 \end{array}\right)$, $\Gamma =\left(\gamma_{ij}\right)\in M_{2n}\left(R\right)$ with $\gamma_{ij}=\mathrm{Tr}[\rho \left(\left(R_{i}-\langle R_{i}\rangle \right)\left(R_{j}-\langle R_{j}\rangle \right)+\left(R_{i}-\langle R_{j}\rangle \right)\left(R_{i}-\langle R_{i}\rangle \right)\right],$ $d=\mathrm{Tr}\left(\rho R\right)\in R^{2n}$, and $R=\left(R_{1},R_{2},\cdots ,R_{2n}\right)=\left({\hat{Q}}_{1},{\hat{P}}_{1},\cdots ,{\hat{Q}}_{n},{\hat{P}}_{n}\right)$. Here, $M_{k}\left(R\right)$ stands for the algebra of all k×k matrices over the real field R. Γ and d above are called respectively the covariance matrix (CM) and the mean of ρ. Note that Γ is real symmetric and satisfies Γ+iJ≥0. In addition, ρ is pure if and only if detΓ=1. If $\rho_{AB}$ is an (n+m)-mode Gaussian state of a bipartite continuous-variable system $H_{A}⊗H_{B}$, its CM Γ can be expressed as $\Gamma =\left(\begin{array}{cc} A & C\\ C^{T} & B \end{array}\right)$, where $A\in M_{2n}\left(R\right)$, $B\in M_{2m}\left(R\right)$ and $C\in M_{2n\times 2m}\left(R\right)$. Particularly, when n=m=1, up to a local Gaussian unitary operation (symplectic at the CM level), Γ has a standard form:

(2) $$\Gamma_{0}=\left(\begin{array}{cc} A_{0} & C_{0}\\ C_{0}^{T} & B_{0} \end{array}\right),\;$$

where $A_{0}=\left(\begin{array}{cc} a & 0\\ 0 & a \end{array}\right)$, $B_{0}=\left(\begin{array}{cc} b & 0\\ 0 & b \end{array}\right)$, $C_{0}=\left(\begin{array}{cc} c & 0\\ 0 & d \end{array}\right)$, a,b≥1, and $ab-1\geq c^{2}\left(d^{2}\right)$. Note that a bipartite state $\rho_{AB}$ is a product state, i.e., $\rho_{AB}=\sigma_{A}⊗\sigma_{B}$, if and only if *C* ($C_{0}$) in its CM (the standard form of its CM) is a zero matrix [8].

For any unitary operator *U* acting on *H*, the unitary operation $\rho \mapsto U\rho U^{†}$ is said to be Gaussian if it sends Gaussian states into Gaussian states, and such a *U* is called a Gaussian unitary operator. It is well known that a unitary operator *U* is Gaussian if and only if $U^{†}RU=SR+m$ for some vector $m\in R^{2n}$ and some S∈Sp(2n,R), the symplectic group of all 2n×2n real matrices. Thus, every Gaussian unitary operator *U* is determined by some affine symplectic map (S,m) acting on the phase space and can be denoted by $U=U_{S,m}$ [35,36].

Now, for any (n+m)-mode state $\rho_{AB}\in S\left(H_{A}⊗H_{B}\right)$, denote by $\rho_{A}={\mathrm{Tr}}_{B}\left(\rho_{AB}\right)$ the reduced state of $\rho_{AB}$. Write:

$$U_{\rho_{AB}}=\left\{U:U\in B\left(H_{A}\right)\;\mathrm{is}\;a\;\mathrm{Gaussian}\;\mathrm{unitary}\;\mathrm{operator}\;\mathrm{satisfying}\;U\rho_{A}U^{†}=\rho_{A}\right\},$$

where B(H) stands for the set of all bounded linear operators acting on *H*. It is obvious that the set $U_{\rho_{AB}}$ is nonempty for any state $\rho_{AB}$; moreover, by [15], for any Gaussian state $\rho_{AB}$, $U_{\rho_{AB}}$ contains many nontrivial Gaussian unitary operators. Thus, we can define a quantum correlation $N_{F}^{G,A}$ by local Gaussian unitary operations for any (n+m)-mode state.

**Definition** **1.** *For any (n+m)-mode state $\rho_{AB}\in S\left(H_{A}⊗H_{B}\right)$, the quantity $N_{F}^{G,A}\left(\rho_{AB}\right)$ with respect to Subsystem A is defined as:*

$$\begin{array}{cc} N_{F}^{G,A}\left(\rho_{AB}\right) & =\underset{U\in U_{\rho_{AB}}}{\sup}C^{2}\left(\rho_{AB},\left(U⊗I\right)\rho_{AB}\left(U^{†}⊗I\right)\right)\\ & =\sup_{U\in U_{\rho_{AB}}}\left\{1-F\left(\rho_{AB},\left(U⊗I\right)\rho_{AB}\left(U^{†}⊗I\right)\right)\right\}, \end{array}$$

*where $F(\rho_{AB},\left(U⊗I\right)\rho_{AB}\left(U^{†}⊗I\right))$ is the Uhlmann fidelity between the pre- and past-measured states, and the supremum is taken over all Gaussian unitary operators $U\in U_{\rho_{AB}}$.*

Similarly, one can define $N_{F}^{G,B}\left(\rho_{AB}\right)$ with respect to Subsystem B. Since the properties of $N_{F}^{G,A}$ and $N_{F}^{G,B}$ are similar, we will focus on discussing the properties of $N_{F}^{G,A}$. For the simplification, we will use $N_{F}^{G}\left(\rho_{AB}\right)$ by omitting the subsystem symbol A (B) unless otherwise specified.

Note that many quantum correlations have the ancilla problem, i.e., when an uncorrelated ancilla System C is appended, the quantities will change due to the local Subsystem C. For example, in [15], a kind of quantum correlation N for (n+m)-mode continuous-variable systems was defined as:

$$N\left(\rho_{AB}\right)=\frac{1}{2}\underset{U}{\sup}{∥\rho_{AB}-\left(U⊗I\right)\rho_{AB}{\left(U⊗I\right)}^{†}∥}_{2}^{2},$$

where the supremum is taken over all unitary operators that maintain $\rho_{A}$ invariant corresponding to Part A. If we append an uncorrelated ancilla System C, the state $\rho_{ABC}=\rho_{AB}⊗\rho_{C}$ can be regarded as a bipartite state with the partition A:BC. After some direct calculations, one has:

$$N\left(\rho_{ABC}\right)=N\left(\rho_{AB}\right)\mathrm{tr}\rho_{C}^{2}.$$

It is obvious that, as long as $\rho_{C}$ is mixed, the quantity N differs arbitrarily due to local ancilla System C. There are other quantum correlations with a similar ancilla problem, such as the quantum correlations proposed in [11,16]. However, our quantity $N_{F}^{G}$ keeps unchanged when appending an ancilla system; that is, we have the following result.

**Theorem** **1.** *$N_{F}^{G}$ is a quantum correlation without the ancilla problem.*

**Proof of Theorem 1.**  Suppose that $\rho_{AB}$ is any bipartite state and C is an uncorrelated ancilla system. Regarding the state $\rho_{ABC}=\rho_{AB}⊗\rho_{C}$ as a bipartite state with the partition A:BC, one has:

$$\begin{array}{cc} & F\left(\rho_{ABC},\left(U⊗I⊗I\right)\rho_{ABC}\right)=F\left(\rho_{AB}⊗\rho_{C},\left(U⊗I\right)\rho_{AB}⊗\rho_{C}\right)\\ = & F\left(\rho_{AB},\left(U⊗I\right)\rho_{AB}\right)\cdot F\left(\rho_{C},\rho_{C}\right)=F\left(\rho_{AB},\left(U⊗I\right)\rho_{AB}\right). \end{array}$$

By Definition 1, we see that $N_{F}^{G}\left(\rho_{ABC}\right)=N_{F}^{G}\left(\rho_{AB}\right)$, completing the proof. □

**Theorem** **2.** *$N_{F}^{G}$ is locally Gaussian unitary invariant, that is, for any (n+m)-mode state $\rho_{AB}$ of continuous-variable system $H_{A}⊗H_{B}$, we have $N_{F}^{G}\left(\left(W⊗V\right)\rho_{AB}\left(W^{†}⊗V^{†}\right)\right)=N_{F}^{G}\left(\rho_{AB}\right)$ for all Gaussian unitary operators $W\in B(H_{A})$ and $V\in B(H_{B})$.*

In the rest of this paper, we mainly consider the case that $\rho_{AB}$ is any Gaussian state.

**Theorem** **3.** *For any (n+m)-mode Gaussian state $\rho_{AB}\in S\left(H_{A}⊗H_{B}\right)$ and any Gaussian channel* Φ *performed on the Subsystem B, we have $N_{F}^{G}\left(\left(I⊗\Phi \right)\rho_{AB}\right)\leq N_{F}^{G}\left(\rho_{AB}\right)$.*

By Definition 1, it is easily checked that $N_{F}^{G}\left(\rho_{AB}\right)=0$ holds for any (n+m)-mode product state $\rho_{AB}$; but we do not know whether the converse is true. The following result reveals that the converse holds for any Gaussian states.

**Theorem** **4.** *For any (n+m)-mode Gaussian state $\rho_{AB}\in S\left(H_{A}⊗H_{B}\right)$, $N_{F}^{G}\left(\rho_{AB}\right)=0$ if and only if $\rho_{AB}$ is a product state.*

Any (1+m)-mode pure Gaussian state can always be brought in the phase-space Schmidt form [37]. The corresponding symplectic transformation S achieving the Schmidt decomposition is the direct sum of two diagonalizing matrices acting on the single-mode and *m*-mode subsystems, respectively, i.e., $S=S_{1}⊕S_{2}$. Suppose Γ is the CM of a (1+m)-mode pure Gaussian state; accordingly, the CM of its phase-space Schmidt form is:

(3) $$\Gamma_{S}=S\Gamma S^{T}=\left(\begin{array}{cccc} \gamma & 0 & \sqrt{\gamma^{2}-1} & 0\\ 0 & \gamma & 0 & -\sqrt{\gamma^{2}-1}\\ \sqrt{\gamma^{2}-1} & 0 & \gamma & 0\\ 0 & -\sqrt{\gamma^{2}-1} & 0 & \gamma \end{array}\right)⊕I_{m-1}\;$$

with γ≥1 the single-mode mixedness factor. We also call $\Gamma_{S}$ the phase-space Schmidt form of Γ. It is clear that the phase-space Schmidt form of a (1+m)-mode pure Gaussian state is the tensor product of a two-mode squeezed state and an (m−1)-mode uncorrelated vacuum state [38].

The following result gives a computation formula of $N_{F}^{G}$ for (1+m)-mode pure Gaussian states.

**Theorem** **5.** *For any (1+m)-mode pure Gaussian state $\rho_{AB}$ with CM* Γ*, we have:*

$$N_{F}^{G}\left(\rho_{AB}\right)=1-\frac{2}{\gamma^{2}+1},$$

*where γ≥1 is the single-mode mixedness factor in the phase-space Schmidt form of* Γ*.*

Recall that a quantum state $\rho_{AB}$ is separable if it belongs to the closed convex hull of the set of all product states $\sigma_{A}⊗\sigma_{B}$ under the trace norm topology. Note that a pure state |ψ〉〈ψ| is separable if and only if it is a product state. The problem of how to quantify entanglement was studied extensively. Generally speaking, an entanglement measure E should meet the following conditions [39]:

(i) E vanishes on separable states;

(ii) E does not increase under local operation and classic communications (LOCC);

(iii) E is locally unitary invariant.

The reader can refer to [1] for more details on entanglement measures.

In [39], a kind of entanglement measure $D_{R}$ for Gaussian states was proposed. It was shown that, for any (1+m)-mode pure Gaussian state $\rho_{AB}$ with CM Γ, $D_{R}\left(\rho_{AB}\right):=\min_{S}\left(1-F\left(\Gamma ,\Gamma^{\prime }\right)\right)$, where *F* stands for the Uhlmann fidelity, $\Gamma^{\prime }=\left(S⊗I_{m}\right)\Gamma {\left(S⊗I_{m}\right)}^{T}$, and S is any traceless symplectic matrix performed on the single-mode. Furthermore, $D_{R}\left(\rho_{AB}\right)=1-\frac{2}{\gamma^{2}+1}$ with γ the single-mode mixedness factor in the phase-space Schmidt form of Γ, which coincides with the quantity $N_{F}^{G}\left(\rho_{AB}\right)$ by Theorem 5. This reveals that $N_{F}^{G}$ is an entanglement measure when it is restricted to (1+m)-mode pure Gaussian states.

According to [40,41,42,43], a bona fide quantum correlation $G_{A}$ for Gaussian states with respect to Subsystem A should satisfy:

(i) $G_{A}\left(\rho_{AB}\right)=0$ if and only if $\rho_{AB}$ is a product state;

(ii) (Locally Gaussian unitary invariant) $G_{A}\left(\left(W⊗V\right)\rho_{AB}\left(W^{†}⊗V^{†}\right)\right)=G_{A}\left(\rho_{AB}\right)$ holds for any Gaussian unitary operators $W\in B(H_{A})$, $V\in B(H_{B})$ and any Gaussian state $\rho_{AB}$;

(iii) (Non-increasing under local Gaussian channels) $G_{A}\left(\left(I⊗\Phi \right)\rho_{AB}\right)\leq G_{A}\left(\rho_{AB}\right)$ holds for any Gaussian channel Φ performed on Subsystem B and any Gaussian state $\rho_{AB}$;

(iv) (Reducing to an entanglement measure for pure states) There exists an entanglement measure E such that $G_{A}(|\psi \rangle \langle \psi |)=E(|\psi \rangle \langle \psi |)$ holds for any bipartite pure state |ψ〉〈ψ|.

By Theorems 2–4, $N_{F}^{G}$ satisfies (i)–(iii); Theorem 5 says that $N_{F}^{G}$ satisfies (iv) for any (1+m)-mode Gaussian pure state. Therefore, at least for (1+m)-mode Gaussian states, $N_{F}^{G}$ is a well-defined Gaussian quantum correlation.

In the rest of this section, let us discuss the question of how to calculate $N_{F}^{G}$. Note that, for any (n+m)-mode Gaussian states ρ and σ with its characteristic function defined as in Equation (1), together with the formula established in [33], we have:

$$F\left(\rho ,\sigma \right)=\mathrm{Tr}\left(\rho \sigma \right)\cdot \Pi_{j=1}^{n+m}\left(\eta_{j}+\sqrt{\eta_{j}^{2}-1}\right),$$

where $\eta_{j}$s (j=1,2,···,n+m) are the symplectic roots of the CM of $\bar{\rho }=\frac{\sqrt{\sigma }\rho \sqrt{\sigma }}{\mathrm{Tr}(\sqrt{\sigma }\rho \sqrt{\sigma })}$. In general, one can use the above fidelity formula to compute $N_{F}^{G}\left(\rho_{AB}\right)$ for any (n+m)-mode Gaussian state $\rho_{AB}$. Due to the theoretical and experimental importance of two-mode symmetric squeezed thermal states (SSTSs), as an example, we give an analytic computation formula for (1+1)-mode SSTSs here.

Recall that SSTSs are Gaussian states whose CMs as in Equation (2) are parameterized by μ and $\bar{n}$ such that $c=-d=2\mu \sqrt{\bar{n}\left(1+\bar{n}\right)}$ and $a=b=1+2\bar{n}$, where μ is a mixing parameter with 0≤μ≤1 and $\bar{n}$ is the mean photon number for each part [44]. Thus, every SSTS can be parameterized as $\rho_{AB}\left(\bar{n},\mu \right)$, and the standard form of its CM is:

$$\Gamma_{0}=\left(\begin{array}{cccc} 1+2\bar{n} & 0 & 2\mu \sqrt{\bar{n}\left(1+\bar{n}\right)} & 0\\ 0 & 1+2\bar{n} & 0 & -2\mu \sqrt{\bar{n}\left(1+\bar{n}\right)}\\ 2\mu \sqrt{\bar{n}\left(1+\bar{n}\right)} & 0 & 1+2\bar{n} & 0\\ 0 & -2\mu \sqrt{\bar{n}\left(1+\bar{n}\right)} & 0 & 1+2\bar{n} \end{array}\right).$$

**Theorem** **6.** *For any (1+1)-mode symmetric squeezed thermal state $\rho_{AB}\left(\bar{n},\mu \right)$, we have:*

(4) $$N_{F}^{G}\left(\rho_{AB}\left(\bar{n},\mu \right)\right)=1-\frac{1}{\left(\sqrt{\Omega }+\sqrt{\Lambda }\right)-\sqrt{{\left(\sqrt{\Omega }+\sqrt{\Lambda }\right)}^{2}-\Upsilon }},\;$$

*where:*

$$\begin{array}{cc} \Upsilon = & \frac{1}{4}{\left[-2{\left(1+2\bar{n}\right)}^{2}+4\bar{n}\left(\bar{n}+1\right)\mu^{2}\right]}^{2},\\ \Omega = & {\left[1-2\bar{n}\left(-2+\mu^{2}\right)+8{\bar{n}}^{3}{\left(-1+\mu^{2}\right)}^{2}+4{\bar{n}}^{4}{\left(\mu^{2}-1\right)}^{2}+2{\bar{n}}^{2}\left(2\mu^{4}-5\mu^{2}+4\right)\right]}^{2},\\ \Lambda = & 16{\bar{n}}^{4}{\left(1+\bar{n}\right)}^{4}{\left(-1+\mu^{2}\right)}^{4}. \end{array}$$

*Moreover,*

(5) $$\lim_{\bar{n}\to \infty }N_{F}^{G}\left(\rho_{AB}\left(\bar{n},\mu \right)\right)=1\;$$

*holds for any μ∈[0,1].*

The proofs of Theorems 2–6 will be given in the Appendix A.

Note that $N_{F}^{G}\left(\rho_{AB}\right)\leq 1$ holds for any Gaussian state $\rho_{AB}$. However, unlike the Gaussian discord case, there is no threshold in terms of $N_{F}^{G}$ for separable states; that is, there is no positive number d<1 such that $N_{F}^{G}\left(\rho_{AB}\right)\leq d$ holds for all separable states $\rho_{AB}$. To see this, recall that a (1+1)-mode Gaussian state $\rho_{AB}$ is separable if and only if ${\tilde{v}}_{-}\geq 1$, where ${\tilde{v}}_{-}$ is the smallest symplectic eigenvalue of the CM of the partial transpose $\rho_{AB}^{T_{B}}$ [45]. For any SSTS $\rho_{AB}\left(\bar{n},\mu \right)$ with CM Γ, we have:

$$\begin{array}{cc} {\tilde{v}}_{-} & =\sqrt{\frac{\left(\det A+\det B-2\det C\right)-\sqrt{{\left(\det A+\det B-2\det C\right)}^{2}-\det\Gamma }}{2}}\\ & =\sqrt{\frac{2{\left(1+2\bar{n}\right)}^{2}+8\bar{n}\left(1+\bar{n}\right)\mu^{2}-\sqrt{64\bar{n}\left(1+\bar{n}\right){\left(\mu +2\bar{n}\mu \right)}^{2}}}{2}}. \end{array}$$

Thus, ${\tilde{v}}_{-}\geq 1$ if and only if either $\bar{n}=0,0\leq \mu \leq 1$ or $0<\bar{n},0\leq \mu \leq \sqrt{\frac{\bar{n}}{\bar{n}+1}}$. Given μ∈(0,1), $\mu <\sqrt{\frac{\bar{n}}{\bar{n}+1}}$ for sufficiently large $\bar{n}$, which guarantees the separability of $\rho_{AB}\left(\bar{n},\mu \right)$. However, by Theorem 6, $\lim_{\bar{n}\to \infty }N_{F}^{G}\left(\rho_{AB}\left(\bar{n},\mu \right)\right)=1$. Therefore, $\sup\{N_{F}^{G}\left(\rho_{AB}\right):\rho_{AB}$ is separable }=1.

## 3. Comparing $N_{F}^{G}$ with Other Quantum Correlations

As is seen, $N_{F}^{G}$ describes the same correlation in Gaussian states as Gaussian quantum discord (GQD) *D*, Gaussian geometric discord (GGD) $D_{G}$, the quantum correlation *Q* proposed in [16], and $N_{F}^{G}$ proposed in [46]. In this section, we will compare $N_{F}^{G}$ with these QCs for two-mode SSTSs in scale. It is clear that, for any SSTS $\rho_{AB}\left(\bar{n},\mu \right)$, we have $N_{F}^{G}\left(\rho_{AB}\left(\bar{n},0\right)\right)=D\left(\rho_{AB}\left(\bar{n},0\right)\right)=D_{G}\left(\rho_{AB}\left(\bar{n},0\right)\right)=Q\left(\rho_{AB}\left(\bar{n},0\right)\right)=N_{F}^{G}\left(\rho_{AB}\left(\bar{n},0\right)\right)=0$. Hence, during the comparison process, we mainly focus our attention on the case μ≠0.

Recall that an *n*-mode Gaussian positive operator-valued measurement (GPOVM) is a collection of positive operators Π={Π(z)} satisfying $\frac{1}{\pi }\int_{z}\Pi \left(z\right)dz=I$, where $\Pi \left(z\right)=D\left(z\right)\tau D^{†}\left(z\right),z\in R^{2n}$ with D(z) the Weyl operators and τ an *n*-mode Gaussian state, which is called the seed of the GPOVM Π [47]. Let $\rho_{AB}$ be an (n+m)-mode Gaussian state and Π={Π(z)} be a GPOVM of the Subsystem A. Denote by $\rho_{B}\left(z\right)=\frac{1}{p(z)}{\mathrm{Tr}}_{A}\left(\rho_{AB}\Pi \left(z\right)⊗I\right)$ the reduced state of the Subsystem B after the GPOVM Π performed on the Subsystem A, where $p\left(z\right)=\mathrm{Tr}(\rho_{AB}\Pi \left(z\right)⊗I)$. Then, the GQD of $\rho_{AB}$ is defined as:

$$D\left(\rho_{AB}\right)=S\left(\rho_{A}\right)-S\left(\rho_{AB}\right)+\underset{\Pi }{\inf}\int p\left(z\right)S\left(\rho_{B}\left(z\right)\right)dz,$$

where the infimum is taken over all GPOVMs Π={Π(z)} performed on Subsystem A and S(ρ)=−Tr(ρlogρ) is the von Neumann entropy [8,9]. It is known that, if the standard form of the CM of a (1+1)-mode Gaussian state $\rho_{AB}$ is as in Equation (2), then:

$$D\left(\rho_{AB}\right)=f\left(\sqrt{{\mathrm{detA}}_{0}}\;\right)-f\left(v_{-}\right)-f\left(v_{+}\right)+f\left(\sqrt{\inf_{\tau }\det E_{\tau }}\;\right),$$

where the infimum takes over all one-mode Gaussian states τ, $f\left(x\right)=\frac{x+1}{2}\log\frac{x+1}{2}-\frac{x-1}{2}\log\frac{x-1}{2}$, $v_{-}$ and $v_{+}$ are the symplectic eigenvalues of the CM of $\rho_{AB}$, and $E_{\tau }=B_{0}-C_{0}{\left(A_{0}+\Gamma_{\tau }\right)}^{-1}C_{0}^{T}$ with $\Gamma_{\tau }$ the CM of τ. Denote by $\alpha =\det A_{0}$, $\beta =\det B_{0}$, $\gamma =\det C_{0}$ and $\delta =\det\Gamma_{0}$; then, we have [8]:

$$\begin{array}{c} \underset{\tau }{\inf}\det E_{\tau }=\left\{\begin{array}{cc} \frac{2\gamma^{2}+\left(\alpha -1\right)\left(\delta -\beta \right)+2\left|\gamma \right|\sqrt{\gamma^{2}+\left(\alpha -1\right)\left(\delta -\beta \right)}}{{\left(\alpha -1\right)}^{2}} & \mathrm{if}\;{\left(\delta -\beta \alpha \right)}^{2}\leq \left(1+\alpha \right)\gamma^{2}\left(\beta +\delta \right),\\ \frac{\beta \alpha -\gamma^{2}+\delta -\sqrt{\gamma^{4}+{\left(\delta -\beta \alpha \right)}^{2}-2\gamma^{2}\left(\beta \alpha +\delta \right)}}{2\alpha } & \mathrm{otherwise}. \end{array}\right. \end{array}$$

Particularly, if $\rho_{AB}=\rho_{AB}\left(\bar{n},\mu \right)$ is an SSTS, one can easily check that ${\left(\delta -\beta \alpha \right)}^{2}\leq \left(1+\alpha \right)\gamma^{2}\left(\beta +\delta \right)$ always holds and $v_{-}=v_{+}=\sqrt{{\left(1+2\bar{n}\right)}^{2}-4\bar{n}\left(1+\bar{n}\right)\mu^{2}}$. In this case, we have:

$$D\left(\rho_{AB}\left(\bar{n},\mu \right)\right)=f\left(1+2\bar{n}\;\right)-2f\left(\sqrt{{\left(1+2\bar{n}\right)}^{2}-4\bar{n}\left(1+\bar{n}\right)\mu^{2}}\right)+f\left(\sqrt{M}\;\right),$$

where:

$$\begin{array}{cc} M= & \frac{1}{\bar{n}\left(1+\bar{n}\right)}[8{\bar{n}}^{3}{\left(-1+\mu^{2}\right)}^{2}+4{\bar{n}}^{4}{\left(-1+\mu^{2}\right)}^{2}+2\mu^{2}\sqrt{N}\\ & +\bar{n}\left(1-2\mu^{2}+2\mu^{4}\right)+{\bar{n}}^{2}\left(5-10\mu^{2}+6\mu^{4}\right)] \end{array}$$

with $N={\left(\bar{n}+3{\bar{n}}^{2}+2{\bar{n}}^{3}\right)}^{2}{\left(-1+\mu^{2}\right)}^{2}$.

In the case μ=1,

$$D\left(\rho_{AB}\left(\bar{n},1\right)\right)=\left(\bar{n}+1\right)\log\left(\bar{n}+1\right)-n\log n=\log\left({\left(1+\frac{1}{\bar{n}}\right)}^{n}\left(\bar{n}+1\right)\right)$$

and hence, $\lim_{\bar{n}\to \infty }D\left(\rho_{AB}\left(\bar{n},1\right)\right)=\infty$. While,

$$N_{F}^{G}\left(\rho_{AB}\left(\bar{n},1\right)\right)=1-\frac{1}{1+2\bar{n}+2{\bar{n}}^{2}}\to 1\;\mathrm{as}\;\bar{n}\to \infty ,$$

therefore, when μ=1, *D* is much greater than $N_{F}^{G}$ for large $\bar{n}$. However, when 0<μ<1, a numerical method reveals that $\lim_{\bar{n}\to \infty }D\left(\rho_{AB}\left(\bar{n},\mu \right)\right)=0$, and by Theorem 6, $\lim_{\bar{n}\to \infty }N_{F}^{G}\left(\rho_{AB}\left(\bar{n},\mu \right)\right)=1$. This means that, for 0<μ<1 and large $\bar{n}$, we have $N_{F}^{G}\left(\rho_{AB}\left(\bar{n},\mu \right)\right)>D\left(\rho_{AB}\left(\bar{n},\mu \right)\right)$. Therefore, when we detect the correlation in SSTS, $N_{F}^{G}$ is much better than *D* for the case 0<μ<1 and large $\bar{n}$.

**Figure 1:**
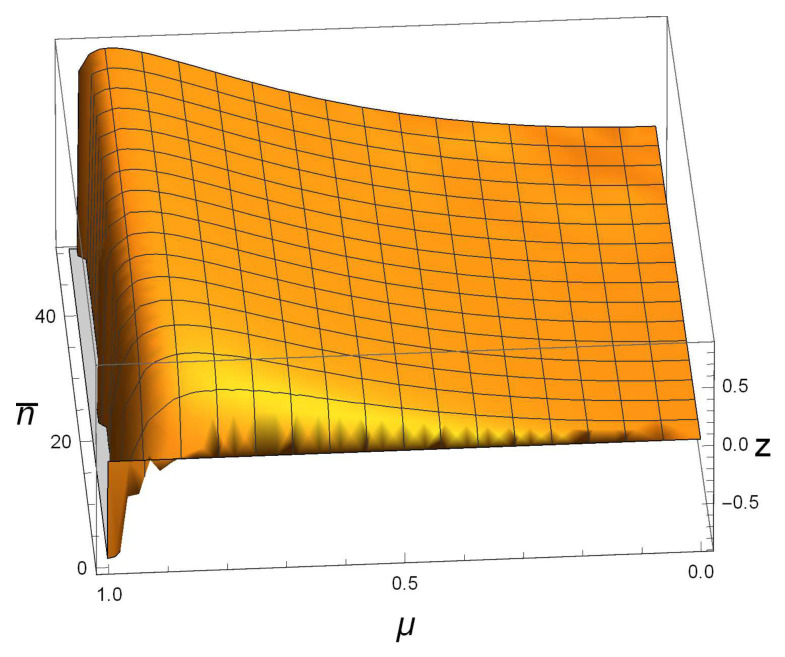
Behavior of z = $N_{F}^{G}\left(\rho_{AB}\left(\bar{n},\mu \right)\right)-D\left(\rho_{AB}\left(\bar{n},\mu \right)\right)$ for symmetric squeezed thermal states (SSTSs) $\rho_{AB}\left(\bar{n},\mu \right)$ with 0≤μ≤1 and $0\leq \bar{n}\leq 50$. When μ is close to one, zis smaller than zero; otherwise, z is bigger than zero.

For small $\bar{n}$, we display the image of $N_{F}^{G}\left(\rho_{AB}\right)-D\left(\rho_{AB}\right)$ for SSTSs in Figure 1 with $0\leq \bar{n}\leq 50$. It shows that $N_{F}^{G}\left(\rho_{AB}\right)>D\left(\rho_{AB}\right)$ for most of the pairs $\left(\bar{n},\mu \right)$, and the inequality is invalid only when μ is in a very small neighborhood of one. For example, by taking an SSTS $\rho_{AB}$ with $\bar{n}=45$ and μ=0.88, we have $D(\rho_{AB}\left(45,0.88\right))\approx 0.05252$, which is too small and difficult to judge whether or not $\rho_{AB}$ is a product state. However, $N_{F}^{G}\left(\rho_{AB}\left(45,0.88\right)\right)\approx 0.864128$ is much bigger than zero, which guarantees that $\rho_{AB}$ is not a product state. In addition, since $N_{F}^{G}\left(\rho_{AB}\left(\bar{n},1\right)\right)=1-\frac{1}{1+2\bar{n}+2{\bar{n}}^{2}}$ is big enough, we conclude that, on the whole, $N_{F}^{G}$ is better than *D* in detecting the correlation in SSTSs, and it is a good choice if we take $h\left(\rho_{AB}\right)=\max\left\{D\left(\rho_{AB}\right),N_{F}^{G}\left(\rho_{AB}\right)\right\}$ as a quantification of this quantum correlation.

In [11], the Gaussian geometric discord (GGD) $D_{G}$ of any two-mode Gaussian state $\rho_{AB}$ is defined by:

$$D_{G}\left(\rho_{AB}\right)=\underset{\Pi^{A}}{\inf}{∥\rho_{AB}-\Pi^{A}\left(\rho_{AB}\right)∥}_{2}^{2},$$

where the infimum runs over all GPOVMs $\Pi^{A}=\left\{\Pi^{A}\left(\alpha \right)\right\}$ of Subsystem *A* and $\Pi^{A}\left(\rho_{AB}\right)=\int {\left(\Pi^{A}\left(\alpha \right)⊗I\right)}^{\frac{1}{2}}\rho_{AB}{\left(\Pi^{A}\left(\alpha \right)⊗I\right)}^{\frac{1}{2}}d^{2}\alpha$. Moreover, it was shown that, for any SSTS $\rho_{AB}$,

$$D_{G}\left(\rho_{AB}\left(\bar{n},\mu \right)\right)=\frac{1}{{\left(1+2\bar{n}\right)}^{2}-4\bar{n}\left(1+\bar{n}\right)\mu^{2}}-\frac{9}{{\left(\sqrt{{\left(1+2\bar{n}\right)}^{2}}+2\sqrt{{\left(1+2\bar{n}\right)}^{2}-3\bar{n}\left(1+\bar{n}\right)\mu^{2}}\right)}^{2}}.$$

For (n+m)-mode continuous-variable systems, in [16], $Q(\rho_{AB})$ is a quantum correlation defined in terms of average distance between the reduced states under the LGPOVMs.

$$Q\left(\rho_{AB}\right)=\underset{\Pi^{A}}{\sup}\int p\left(\alpha \right){∥\rho_{B}-\rho_{B}^{\left(\alpha \right)}∥}_{2}^{2}d_{\alpha }^{2m},$$

where the supremum is taken over all GPOVMs $\Pi^{A}=\left\{\Pi^{A}\left(\alpha \right)\right\}$ on the subsystem $H_{A}$, $\rho_{B}={\mathrm{Tr}}_{A}\left(\rho_{AB}\right)$, $p\left(\alpha \right)=\mathrm{Tr}[\left(\Pi^{A}\left(\alpha \right)⊗I_{B}\right)\rho_{AB}]$, and $\rho_{B}^{\left(\alpha \right)}=\frac{1}{p(\alpha )}{\mathrm{Tr}}_{A}\left[{\left(\Pi^{A}\left(\alpha \right)⊗I_{B}\right)}^{\frac{1}{2}}\rho_{AB}{\left(\Pi^{A}\left(\alpha \right)⊗I_{B}\right)}^{\frac{1}{2}}\right]$.

For any SSTS $\rho_{AB}\left(\bar{n},\mu \right)$,^16^] provided an analytical formula as:

$$Q\left(\rho_{AB}\left(\bar{n},\mu \right)\right)=\frac{1}{1+2\bar{n}\left(1-\mu^{2}\right)}-\frac{1}{1+2\bar{n}}.$$

**Figure 2:**
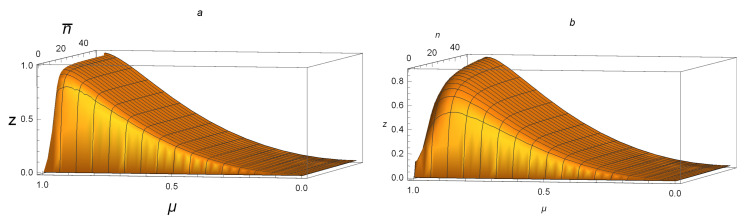
For SSTSs $\rho_{AB}\left(\bar{n},\mu \right)$ with 0≤μ≤1 and $0\leq \bar{n}\leq 50$: (**a**) z = $N_{F}^{G}\left(\rho_{AB}\left(\bar{n},\mu \right)\right)-D_{G}\left(\rho_{AB}\left(\bar{n},\mu \right)\right)$; (**b**) z = $N_{F}^{G}\left(\rho_{AB}\left(\bar{n},\mu \right)\right)-Q\left(\rho_{AB}\left(\bar{n},\mu \right)\right)$. Both figures are above the $\bar{n}o\mu$ plane, and the peaks in both figures are near one and 0.8, respectively.

Figure 2a,b shows that $N_{F}^{G}\left(\rho_{AB}\right)>D_{G}\left(\rho_{AB}\right)$ and $N_{F}^{G}\left(\rho_{AB}\left(\bar{n},\mu \right)\right)>Q\left(\rho_{AB}\left(\bar{n},\mu \right)\right)$ for all SSTSs with 0<μ≤1 and $0\leq \bar{n}\leq 50$. For example, taking $\bar{n}=45$ and μ=0.88, one sees that $D_{G}\left(\rho_{AB}\left(45,0.88\right)\right)\approx 0.00033\approx 0$, $Q(\rho_{AB}\left(45,0.88\right))\approx 0.02750\approx 0$, while $N_{F}^{G}\left(\rho_{AB}\left(45,0.88\right)\right)\approx 0.86413\gg 0$. This suggests that $N_{F}^{G}\left(\rho_{AB}\right)$ is better at detecting whether or not a state is a product state with small $\bar{n}$.

When μ≠1, it is clear that $\lim_{\bar{n}\to \infty }D_{G}\left(\rho_{AB}\left(\bar{n},\mu \right)\right)=\lim_{\bar{n}\to \infty }Q\left(\rho_{AB}\left(\bar{n},\mu \right)\right)=0$. Hence, $N_{F}^{G}\left(\rho_{AB}\right)$ is much greater than both $D_{G}\left(\rho_{AB}\right)$ and $Q(\rho_{AB})$ for those SSTSs $\rho_{AB}\left(\bar{n},\mu \right)$ with large mean photon number $\bar{n}$ and 0<μ<1. To be specific, let $\left(\bar{n},\mu \right)=\left(1000,0.7\right)$; one has $N_{F}^{G}\left(\rho_{AB}\left(1000,0.7\right)\right)=0.54372$, while $D_{G}\left(\rho_{AB}\left(1000,0.7\right)\right)=1.5478\times 10^{-7}$ and $Q\left(\rho_{AB}\left(1000,0.7\right)\right)=4.7968\times 10^{-4}$ are too small to ensure that such a state is not a product state.

Now, consider the case μ=1. One has:

$$D_{G}\left(\rho_{AB}\left(\bar{n},1\right)\right)=1-\frac{9}{{\left(1+2\bar{n}+2\sqrt{1+\bar{n}+{\bar{n}}^{2}}\right)}^{2}};$$

$$Q\left(\rho_{AB}\left(\bar{n},1\right)\right)=1-\frac{1}{1+2\bar{n}}.$$

It is easy to verify that $N_{F}^{G}\left(\rho_{AB}\left(\bar{n},1\right)\right)>D_{G}\left(\rho_{AB}\left(\bar{n},1\right)\right)>Q\left(\rho_{AB}\bar{n},1\right))$, even though $\lim_{\bar{n}\to \infty }N_{F}^{G}\left(\rho_{AB}\left(\bar{n},1\right)\right)=\lim_{\bar{n}\to \infty }D_{G}\left(\rho_{AB}\left(\bar{n},1\right)\right)=\lim_{\bar{n}\to \infty }Q\left(\rho_{AB}\left(\bar{n},1\right)\right)=1$.

The discussions above together with Figure 2a,b suggest that $N_{F}^{G}\left(\rho_{AB}\right)>D_{G}\left(\rho_{AB}\right)$ and $N_{F}^{G}\left(\rho_{AB}\left(\bar{n},\mu \right)\right)>Q\left(\rho_{AB}\left(\bar{n},\mu \right)\right)$ hold for all SSTSs. The numerical analysis supports these assertions.

In [46], we proposed a quantum correlation $N_{F}^{G}$ for (n+m)-mode Gaussian systems based on another form of fidelity $F\left(\rho ,\sigma \right)=\frac{{\left(\mathrm{Tr}\rho \sigma \right)}^{2}}{\mathrm{Tr}\left(\rho^{2}\right)\mathrm{Tr}\left(\sigma^{2}\right)}$ introduced in [48], which is defined as:

$$N_{F}^{G}\left(\rho_{AB}\right)=\underset{U\in U_{\rho_{AB}}}{\sup}\left\{1-F\left(\rho_{AB},\left(U⊗I\right)\rho_{AB}{\left(U⊗I\right)}^{†}\right)\right\}.$$

The quantity $N_{F}^{G}$ has several similar properties as $N_{F}^{G}$, but is easier to calculate. Particularly, for SSTS $\rho_{AB}\left(\bar{n},\mu \right)$, one has:

$$N_{F}^{G}\left(\rho_{AB}\left(\bar{n},\mu \right)\right)=1-\frac{{\left({\left(1+2\bar{n}\right)}^{2}-4\bar{n}\left(1+\bar{n}\right)\mu^{2}\right)}^{2}}{{\left({\left(1+2\bar{n}\right)}^{2}-2\bar{n}\left(1+\bar{n}\right)\mu^{2}\right)}^{2}}.$$

**Figure 3:**
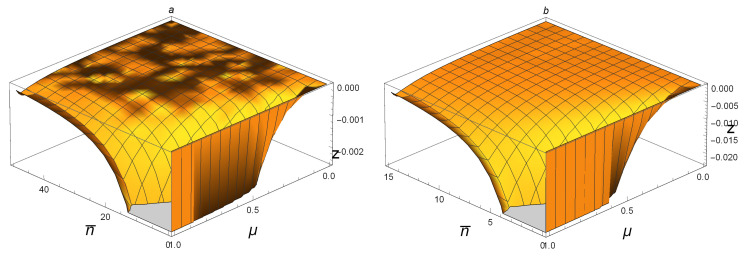
For SSTSs $\rho_{AB}\left(\bar{n},\mu \right)$: (**a**) z = $N_{F}^{G}\left(\rho_{AB}\left(\bar{n},\mu \right)\right)-N_{F}^{G}\left(\rho_{AB}\left(\bar{n},\mu \right)\right)$ with 0≤μ≤1 and $0\leq \bar{n}\leq 50$; (**b**) z = $N_{F}^{G}\left(\rho_{AB}\left(\bar{n},\mu \right)\right)-N_{F}^{G}\left(\rho_{AB}\left(\bar{n},\mu \right)\right)$ with 0≤μ≤1 and $0\leq \bar{n}\leq 15$. The gray areas in the $\bar{n}o\mu$ plane are cutoffs caused by the drawing software.

**Figure 4:**
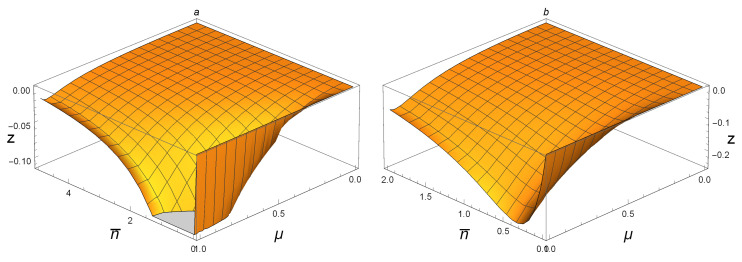
(**a**) z = $N_{F}^{G}\left(\rho_{AB}\left(\bar{n},\mu \right)\right)-N_{F}^{G}\left(\rho_{AB}\left(\bar{n},\mu \right)\right)$ with 0≤μ≤1 and $0\leq \bar{n}\leq 5$; (**b**) z = $N_{F}^{G}\left(\rho_{AB}\left(\bar{n},\mu \right)\right)-N_{F}^{G}\left(\rho_{AB}\left(\bar{n},\mu \right)\right)$ with 0≤μ≤1 and $0\leq \bar{n}\leq 2$; for SSTSs $\rho_{AB}\left(\bar{n},\mu \right)$, respectively.

**Figure 5:**
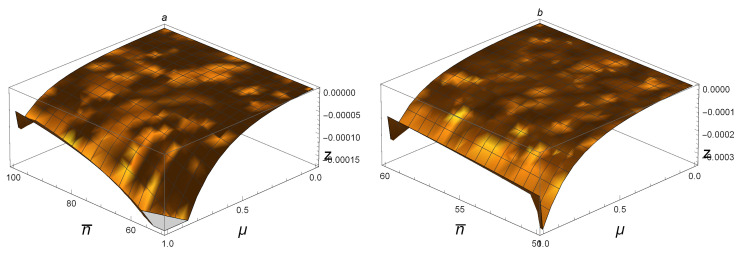
Comparing $N_{F}^{G}$ with $N_{F}^{G}$ for SSTSs $\rho_{AB}\left(\bar{n},\mu \right)$ by: (**a**) z = $N_{F}^{G}\left(\rho_{AB}\left(\bar{n},\mu \right)\right)-N_{F}^{G}\left(\rho_{AB}\left(\bar{n},\mu \right)\right)$ with 0≤μ≤1 and $0\leq \bar{n}\leq 100$; (**b**) z = $N_{F}^{G}\left(\rho_{AB}\left(\bar{n},\mu \right)\right)-N_{F}^{G}\left(\rho_{AB}\left(\bar{n},\mu \right)\right)$ with 0≤μ≤1 and $50\leq \bar{n}\leq 60$. Numerically, the difference is very small.

In order to get the full graph of $z=N_{F}^{G}-N_{F}^{G}$ for SSTSs, we have to use six figures since there exist cutoffs caused by the drawing software. In Figure 3a, we plot the function of $N_{F}^{G}\left(\rho_{AB}\right)-N_{F}^{G}\left(\rho_{AB}\right)$ for SSTSs $\rho_{AB}$ with $0\leq \bar{n}\leq 50$ and 0<μ≤1. It is clear that, when $15\leq \bar{n}\leq 50$, one has $0\leq N_{F}^{G}\left(\rho_{AB}\right)-N_{F}^{G}\left(\rho_{AB}\right)\leq 0.003$. Figure 3b shows that $0\leq N_{F}^{G}\left(\rho_{AB}\right)-N_{F}^{G}\left(\rho_{AB}\right)\leq 0.025$ if $5\leq \bar{n}\leq 15$ and 0<μ≤1. The cases when $0\leq \bar{n}\leq 5$ and $0\leq \bar{n}\leq 2$ are shown in Figure 4a and Figure 4b, respectively. Accordingly, one can tell that $0\leq N_{F}^{G}\left(\rho_{AB}\right)-N_{F}^{G}\left(\rho_{AB}\right)\leq 0.12$ if $2\leq \bar{n}\leq 5$ and $0\leq N_{F}^{G}\left(\rho_{AB}\right)-N_{F}^{G}\left(\rho_{AB}\right)\leq 0.25$ when $0\leq \bar{n}\leq 2$. Hence, for any Gaussian state $\rho_{AB}\left(\bar{n},\mu \right)$ with $0\leq \bar{n}\leq 50$ and 0<μ≤1, one can conclude that $0\leq N_{F}^{G}\left(\rho_{AB}\right)-N_{F}^{G}\left(\rho_{AB}\right)\leq 0.12$. When the average photon number gets bigger, as shown in Figure 5a,b, one has $0\leq N_{F}^{G}\left(\rho_{AB}\right)-N_{F}^{G}\left(\rho_{AB}\right)\leq 0.0002$ if $60\leq \bar{n}\leq 100$; when $50\leq \bar{n}\leq 60$, it holds that $0\leq N_{F}^{G}\left(\rho_{AB}\right)-N_{F}^{G}\left(\rho_{AB}\right)\leq 0.0004$. This means that, though $N_{F}^{G}\left(\rho_{AB}\left(\bar{n},\mu \right)\right)<N_{F}^{G}\left(\rho_{AB}\left(\bar{n},\mu \right)\right)$ for small $\bar{n}$, the difference between them is very small. For large $\bar{n}$, as $\lim_{\bar{n}\to \infty }N_{F}^{G}\left(\rho \left(\bar{n},\mu \right)\right)=1-\frac{{\left(1-\mu^{2}\right)}^{2}}{{\left(1-\frac{1}{2}\mu^{2}\right)}^{2}}<1$, while $\lim_{\bar{n}\to \infty }N_{F}^{G}\left(\rho \left(\bar{n},\mu \right)\right)=1$, we see that $N_{F}^{G}\left(\rho_{AB}\left(\bar{n},\mu \right)\right)>N_{F}^{G}\left(\rho_{AB}\left(\bar{n},\mu \right)\right)$ for large $\bar{n}$ and 0<μ<1, and the difference between them may be very big. This can be seen by the fact $\lim_{\mu \to 0}\left(1-\frac{{\left(1-\mu^{2}\right)}^{2}}{{\left(1-\frac{1}{2}\mu^{2}\right)}^{2}}\right)=0$. For the special case μ=1, we have:

$$N_{F}^{G}\left(\rho \left(\bar{n},1\right)\right)=1-\frac{1}{{\left(1+2\bar{n}+2{\bar{n}}^{2}\right)}^{2}}\to 1\;\mathrm{as}\;\;\bar{n}\to \infty .$$

It is clear that $N_{F}^{G}\left(\rho_{AB}\left(\bar{n},1\right)\right)$ is bigger than $N_{F}^{G}\left(\rho_{AB}\left(\bar{n},1\right)\right)$ for every $\bar{n}$, but the difference between them is very small.

In summary, we can conclude that, if 0<μ<1, on the whole, the behavior of $N_{F}^{G}$ is better than $N_{F}^{G}$ in detecting correlation contained in an SSTS.

From the above analysis, for Gaussian states, the quantity $N_{F}^{G}$ describes the same quantum correlation as *D*, $D_{G}$, and *Q*. Furthermore, the quantity $N_{F}^{G}$ we proposed has no ancilla problem; for a fixed SSTS $\rho_{AB}\left(\bar{n},\mu \right)$, $N_{F}^{G}\left(\rho_{AB}\left(\bar{n},\mu \right)\right)$ is bigger than that of $D_{G}$ and *Q* in scale, which makes it better at detecting non-product Gaussian states; taking into consideration the physic resources consumed during the measurement process, $N_{F}^{G}$ consumes less and simpler resources compared with *D*, $D_{G}$, and *Q*, since we use only part of unitary measurements, while the other three use all GPOVMs. Therefore, the quantity $N_{F}^{G}$ is a reasonable choice to quantify such Gaussian quantum correlations.

Also notice that, in [14], for any two-mode Gaussian state $\rho_{AB}$ with CM Γ, the quantum correlation named Gaussian response of discord ($GD_{R}^{x}$) is proposed, where the index *x* stands for trace, Hellinger, or Bures distance. Precisely, with metrics $d_{tr}\left(\rho ,\sigma \right)={∥\rho -\sigma ∥}_{1}$, $d_{Hel}=\sqrt{\mathrm{tr}{\left(\sqrt{\rho }-\sqrt{\sigma }\right)}^{2}}$ and $d_{Bu}\left(\rho ,\sigma \right)=\sqrt{2(1-\sqrt{F(\rho ,\sigma )})}$,

$$GD_{R}^{x}\left(\rho_{AB}\right):=\min_{S_{A}}N_{x}d_{x}^{2}\left(\rho_{AB},{\tilde{\rho }}_{AB}\right),$$

where the minimum is taken over all local unitaries of which the corresponding local symplectic transformations $S_{A}$ are traceless, the normalization factors $N_{tr}=\frac{1}{4}$ and $N_{Hell}=N_{Bu}=\frac{1}{2}$, and ${\tilde{\rho }}_{AB}$ is the transformed state with CM $\left(S_{A}⊗I\right)\Gamma {\left(S_{A}⊗I\right)}^{T}$. Now, if the sine metric is applied, we can also get a kind of Gaussian response of discord:

$$GD_{R}^{Sin}\left(\rho_{AB}\right):=\min_{S_{A}}C^{2}\left(\rho_{AB},{\tilde{\rho }}_{AB}\right).$$

One can verify that $GD_{R}^{Sin}$ is an alternative quantification of the quantum correlation for Gaussian states. Then, it is interesting to consider the relation between $N_{F}^{G}$ and $GD_{R}^{Sin}$. We claim that $N_{F}^{G}\neq GD_{R}^{Sin}$ even for the two-mode case. To see this, consider the two-mode Gaussian state $\rho_{AB}=\rho \left(a,b,c,d\right)$ with CM $\Gamma \left(a,b,c,d\right)=\left(\begin{array}{cc} A & C\\ C^{T} & B \end{array}\right)$, where $A=\left(\begin{array}{cc} a & 0\\ 0 & a \end{array}\right)$, $B=\left(\begin{array}{cc} b & 0\\ 0 & b \end{array}\right)$ and $C=\left(\begin{array}{cc} c & 0\\ 0 & d \end{array}\right)$ satisfying $ab\geq c^{2}\;\left(d^{2}\right)$. Clearly,

$$U_{\rho_{AB}}=\left\{U_{S_{\theta }}:S_{\theta }=\left(\begin{array}{cc} \cos\theta & \sin\theta \\ -\sin\theta & \cos\theta \end{array}\right),\theta \in \left[0,\frac{\pi }{2}\right]\right\}$$

and the set of all traceless symplectic matrices is:

$$S=\{S_{\alpha ,\beta ,\gamma }=\left(\begin{array}{cc} \gamma & \alpha +\beta \\ \alpha -\beta & -\gamma \end{array}\right):\alpha^{2}+\gamma^{2}+1=\beta^{2}\}.$$

Let:

$$f\left(a,b,c,d,\theta \right)=C^{2}\left(\rho_{AB},\left(U_{S_{\theta }}⊗I\right)\rho_{AB}{\left(U_{S_{\theta }}⊗I\right)}^{†}\right)$$

and:

$$g\left(a,b,c,d,\alpha ,\beta ,\gamma \right)=C^{2}\left(\rho_{AB},\left(U_{S_{\alpha ,\beta ,\gamma }}⊗I\right)\rho_{AB}{\left(U_{S_{\alpha ,\beta ,\gamma }}⊗I\right)}^{†}\right).$$

Then:

$$N_{F}^{G}\left(\rho_{AB}\right)=\max_{0\leq \theta \leq \pi /2}f\left(a,b,c,d,\theta \right)$$

and:

$$GD_{R}^{Sin}\left(\rho_{AB}\right)=\min_{\left(\alpha ,\beta ,\gamma \right):\alpha^{2}+\gamma^{2}+1=\beta^{2}}g\left(a,b,c,d,\alpha ,\beta ,\gamma \right).$$

Picking (a,b,c,d)=(2000,2,62,2), one has $N_{F}^{G}\left(\rho_{AB}\right)\approx 0.5185$ with the maximum value achieved at θ=π/2; while $GD_{R}^{Sin}\left(\rho_{AB}\right)\approx 0.3024$ with the minimum achieved at (α,β)≈(−0.9026002466,1.3471033029). Therefore, $N_{F}^{G}\left(\rho_{AB}\right)\neq GD_{R}^{Sin}\left(\rho_{AB}\right)$. However, for two-mode pure Gaussian states, we find that $N_{F}^{G}=GD_{R}^{Sin}$ because the extremal single-mode operation at the symplectic level coincides for the two quantities, i.e., $S_{A}=\left(\begin{array}{cc} 0 & 1\\ -1 & 0 \end{array}\right).$

## 4. Conclusions

Based on the Uhlmann fidelity *F*, a quantum correlation $N_{F}^{G,A}$ is proposed in terms of local Gaussian unitary operations for any states in (n+m)-mode continuous-variable systems. $N_{F}^{G,A}$ has several nice features: $N_{F}^{G,A}$ is a quantum correlation without the ancilla problem; is local Gaussian unitary invariant; is zero for product states and vice versa for Gaussian states; is monotonically non-increasing under Gaussian quantum channels acting on Subsystem B; and reduces to an entanglement measure for (1+m)-mode pure Gaussian states. However, evaluating $N_{F}^{G,A}$ is also difficult. Computation formulas for any (1+1)-mode symmetric squeezed thermal states and (1+m)-mode pure Gaussian states are established. For general (n+m)-mode Gaussian states, an approach to evaluate the quantity $N_{F}^{G,A}$ is provided. Comparing the behavior of $N_{F}^{G,A}$ in scale with Gaussian quantum discord *D*, Gaussian geometric discord $D_{G}$, and quantum correlations *Q* and $N_{F}^{G}$ for two-mode symmetric squeezed thermal states reveals that $N_{F}^{G,A}$ has some advantages in detecting quantum correlation in Gaussian states.

## Appendix A

In this appendix, we present our proofs of Theorems 2–6.

**Proof of Theorem 2.** For any (n+m)-mode state $\rho_{AB}\in S\left(H_{A}⊗H_{B}\right)$, by Definition 1, we have:

$$\begin{array}{cc} N_{F}^{G}\left(\rho_{AB}\right) & =\sup_{U\in U_{\rho_{AB}}}C^{2}\left(\rho_{AB},\left(U⊗I\right)\rho_{AB}\left(U^{†}⊗I\right)\right)\\ & =\sup_{U\in U_{\rho_{AB}}}\left\{1-F\left(\rho_{AB},\left(U⊗I\right)\rho_{AB}\left(U^{†}⊗I\right)\right)\right\}\\ & =1-\inf_{U\in U_{\rho_{AB}}}F\left(\rho_{AB},\left(U⊗I\right)\rho_{AB}\left(U^{†}⊗I\right)\right). \end{array}$$

For any Gaussian unitary operators $W\in B(H_{A})$ and $V\in B(H_{B})$, write $\sigma_{AB}=\left(W⊗V\right)\rho_{AB}\left(W^{†}⊗V^{†}\right)$. To prove that $N_{F}^{G}\left(\rho_{AB}\right)$ is locally Gaussian unitary invariant, we have to check that:

(A1) $$\underset{U\in U_{\rho_{AB}}}{\inf}F\left(\rho_{AB},\left(U⊗I\right)\rho_{AB}\left(U^{†}⊗I\right)\right)=\underset{U^{\prime }\in U_{\sigma_{AB}}}{\inf}F\left(\sigma_{AB},\left(U^{\prime }⊗I\right)\sigma_{AB}\left(U^{\prime †}⊗I\right)\right).\;$$

Clearly, $\sigma_{A}={\mathrm{Tr}}_{B}\left(\sigma_{AB}\right)=W\rho_{A}W^{†}$. For any Gaussian unitary operator $U^{\prime }\in U_{\sigma_{AB}}$, let $U={\hat{U}}^{†}=W^{†}U^{\prime }W$. It is obvious that *U* is a Gaussian unitary operator satisfying $U\rho_{A}U^{†}=W^{†}U^{\prime }W\rho_{A}W^{†}U^{\prime †}W=W^{†}\sigma_{A}W=\rho_{A}$. Therefore, $U\in U_{\rho_{AB}}$. On the other hand, for any $U\in U_{\rho_{AB}}$, we have $WUW^{†}\in U_{\sigma_{AB}}$. It follows that $W^{†}U_{\sigma_{AB}}W=U_{\rho_{AB}}$. Note that:

$$\begin{array}{cc} & F(\sigma_{AB},\left(U^{\prime }⊗I\right)\sigma_{AB}\left(U^{\prime †}⊗I\right))\\ = & \mathrm{Tr}\sqrt{\sqrt{\left(U^{\prime }⊗I\right)\sigma_{AB}\left(U^{\prime †}⊗I\right)}\sigma_{AB}\sqrt{\left(U^{\prime }⊗I\right)\sigma_{AB}\left(U^{\prime †}⊗I\right)}}\\ = & \mathrm{Tr}\sqrt{\left(U^{\prime }⊗I\right)\sqrt{\sigma_{AB}}\left(U^{\prime †}⊗I\right)\sigma_{AB}\left(U^{\prime }⊗I\right)\sqrt{\sigma_{AB}}\left(U^{\prime †}⊗I\right)}\\ = & \mathrm{Tr}\left(U^{\prime }⊗I\right)\left(W⊗V\right)\sqrt{\sqrt{\rho_{AB}}\left(W^{†}U^{\prime †}W⊗I\right)\rho_{AB}\left(W^{†}U^{\prime }W⊗I\right)\sqrt{\rho_{AB}}}\left(W^{†}⊗V^{†}\right)\left(U^{\prime †}⊗I\right)\\ = & \mathrm{Tr}\sqrt{\sqrt{\rho_{AB}}\left({\hat{U}}^{†}⊗I\right)\rho_{AB}\left(\hat{U}⊗I\right)\sqrt{\rho_{AB}}}=F\left(\left(U⊗I\right)\rho_{AB}\left(U^{†}⊗I\right),\rho_{AB}\right)\\ = & F(\rho_{AB},\left(U⊗I\right)\rho_{AB}\left(U^{†}⊗I\right)). \end{array}$$

This implies that Equation (A1) is true. The proof is completed. ☐

**Proof of Theorem 3.** Suppose that Φ is a local Gaussian channel performed on System B. Note that, for any local Gaussian unitary operation U performed on System A (i.e., there is some Gaussian unitary operator *U* so that $U\rho =U\rho U^{†}$ for each state ρ), we have:

(I⊗Φ)∘(U⊗I)=U⊗Φ=(U⊗I)∘(I⊗Φ).

So, for any Gaussian state $\rho_{AB}\in S\left(H_{A}⊗H_{B}\right)$,

$$\begin{array}{cc} N_{F}^{G}\left(\left(I⊗\Phi \right)\rho_{AB}\right) & =\sup_{U\in U_{\rho_{AB}}}C^{2}\left(\left(I⊗\Phi \right)\rho_{AB},\left(U⊗I\right)\left(I⊗\Phi \right)\rho_{AB}\left(U^{†}⊗I\right)\right)\\ & =\sup_{U\in U_{\rho_{AB}}}\left\{1-F\left(\left(I⊗\Phi \right)\rho_{AB},\left(U⊗I\right)\left(I⊗\Phi \right)\rho_{AB}\left(U^{†}⊗I\right)\right)\right\}\\ & =\sup_{U\in U_{\rho_{AB}}}\left\{1-F\left(\left(I⊗\Phi \right)\rho_{AB},\left(I⊗\Phi \right)\left(U⊗I\right)\rho_{AB}\left(U^{†}⊗I\right)\right)\right\}\\ & \leq \sup_{U\in U_{\rho_{AB}}}\left\{1-F\left(\rho_{AB},\left(U⊗I\right)\rho_{AB}\left(U^{†}⊗I\right)\right)\right\}\\ & =N_{F}^{G}\left(\rho_{AB}\right), \end{array}$$

where the third inequality is due to the monotonicity of the fidelity F [25]. ☐

**Proof of Theorem 4.** By Definition 1, the “if” part is apparent. It is sufficient to check the “only if” part. Assume that $\rho_{AB}$ is any (n+m)-mode Gaussian state so that $N_{F}^{G}\left(\rho_{AB}\right)=0$. Since the mean of any Gaussian state can be transformed to zero by local Gaussian unitary operations, by Theorem 2, we can always assume that the mean of $\rho_{AB}$ is zero and has CM: $\Gamma =\left(\begin{array}{cc} A & C\\ C^{T} & B \end{array}\right)$. By [35], the reduced states $\rho_{A}$ and $\rho_{B}$ have CMs *A* and *B*, respectively. According to the Williamson theorem, there exists a symplectic matrix $S_{0}$ such that $S_{0}AS_{0}^{T}={⊕}_{i=1}^{n}v_{i}I$ and $U_{0}\rho_{A}U_{0}^{†}={⊗}_{i=1}^{n}\rho_{i}$, where $U_{0}=U_{S_{0},0}$ and $\rho_{i}$s are some thermal states. Write $\sigma_{AB}=\left(U_{0}⊗I\right)\rho_{AB}\left(U_{0}^{†}⊗I\right)$. It follows from Theorem 2 that $N_{F}^{G}\left(\sigma_{AB}\right)=N_{F}^{G}\left(\rho_{AB}\right)=0$. Obviously, $\sigma_{AB}$ has zero mean and CM of the form:

$$\Gamma^{\prime }=\left(\begin{array}{cc} {⊕}_{i}^{n}v_{i}I & C^{\prime }\\ C^{\prime T} & B^{\prime } \end{array}\right).$$

Note that, by [15] and [35,36], for Gaussian unitary operator $U_{S,m}\in B\left(H_{A}\right)$ with m=0 and $S={⊕}_{i=1}^{n}S_{\theta_{i}}$, where: $S_{\theta_{i}}=\left(\begin{array}{cc} \cos\theta_{i} & \sin\theta_{i}\\ -\sin\theta_{i} & \cos\theta_{i} \end{array}\right)$ for some $\theta_{i}\in \left[0,\frac{\pi }{2}\right]$, we have $U_{S,m}\sigma_{A}U_{S,m}^{†}=\sigma_{A}={\mathrm{Tr}}_{B}\left(\sigma_{AB}\right)$, that is $U_{S,m}\in U_{\sigma_{AB}}$. By Definition 1, $N_{F}^{G}\left(\sigma_{AB}\right)=0$ entails $F(\sigma_{AB},\left(U⊗I\right)\sigma_{AB}\left(U^{†}⊗I\right))=1$ for all $U\in U_{\sigma_{AB}}$. Thus, $F(\sigma_{AB},\left(U_{S,m}⊗I\right)\sigma_{AB}\left(U_{S,m}^{†}⊗I\right))=1$, which forces:

$$\sigma_{AB}=\left(U_{S,m}⊗I\right)\sigma_{AB}\left(U_{S,m}^{†}⊗I\right).$$

Hence, $\sigma_{AB}$ and $\left(U_{S,m}⊗I\right)\sigma_{AB}\left(U_{S,m}^{†}⊗I\right)$ have the same CMs, that is,

$$\begin{array}{c} \left(\begin{array}{cc} {⊕}_{i=1}^{n}v_{i}I & C^{\prime }\\ C^{\prime T} & B^{\prime } \end{array}\right)=\left(\begin{array}{cc} {⊕}_{i=1}^{n}v_{i}I & SC^{\prime }\\ C^{\prime T}S^{T} & B^{\prime } \end{array}\right). \end{array}$$

If we take $\theta_{i}\in \left(0,\frac{\pi }{2}\right)$ for each *i*, then I−S is an invertible matrix. Therefore, we must have $C^{\prime }=0$; that is, $\sigma_{AB}$ is a product state. It follows that $\rho_{AB}=\left(U_{0}^{†}⊗I\right)\sigma_{AB}\left(U_{0}⊗I\right)$ is also a product state. ☐

**Proof of Theorem 5.** By Theorem 2, $N_{F}^{G}$ is locally Gaussian unitary invariant. Therefore, for any (1+m)-mode pure Gaussian state |ψ〉〈ψ|, it is sufficient to assume that the state is in phase-space Schmidt form. Thus, its CM Γ has the form as in Equation (3) with single-mode mixedness factor γ≥1. Accordingly, the CM of the transformed pure state $\left(U_{1}⊗I_{m}\right)\left|\psi \rangle \langle \psi \right|\left(U_{1}^{†}⊗I_{m}\right)$ is $\left(S_{1}⊕I_{m}\right)\Gamma {\left(S_{1}⊕I_{m}\right)}^{T}$, where $U_{1}$ represents the Gaussian unitary operator performed on the single mode and $I_{m}$ stands for the identity on the other *m*-mode. By the definition of $N_{F}^{G}$, $U_{1}$ keeps the single-mode invariant, which implies $S_{1}=S_{\theta }=\left(\begin{array}{cc} \cos\theta & \sin\theta \\ -\sin\theta & \cos\theta \end{array}\right)$ with $\theta \in [0,\frac{\pi }{2}]$. Notice that for *n*-mode pure Gaussian states ρ and σ with CM $\Gamma_{\rho }$ and $\Gamma_{\sigma }$, respectively, the Uhlmann fidelity between them can be computed as [39,49]:

$$F\left(\rho ,\sigma \right)=\frac{2^{n}}{\sqrt{\det(\Gamma_{\rho }+\Gamma_{\sigma })}}.$$

Applying this formula and after some straight-forward calculations, one gets:

$$\det\left(\Gamma +\left(S_{1}⊕I_{m}\right)\Gamma {\left(S_{1}⊕I_{m}\right)}^{T}\right)=2^{2n-2}{\left(1+\gamma^{2}-\left(\gamma^{2}-1\right)\cos\theta \right)}^{2}.$$

Hence, $N_{F}^{G}(|\psi \rangle \langle \psi |)=\max_{\theta \in [0,\frac{\pi }{2}]}\left(1-\frac{2}{1+\gamma^{2}-\left(\gamma^{2}-1\right)\cos\theta }\right)=1-\frac{2}{1+\gamma^{2}}$. ☐

**Proof of Theorem 6.** Assume that $\rho_{AB}=\rho_{AB}\left(\bar{n},\mu \right)$ is any (1+1)-mode SSTS with CM:

$$\Gamma_{0}=\left(\begin{array}{cccc} a & 0 & c & 0\\ 0 & a & 0 & d\\ c & 0 & b & 0\\ 0 & d & 0 & b \end{array}\right)=\left(\begin{array}{cccc} 1+2\bar{n} & 0 & 2\mu \sqrt{\bar{n}\left(1+\bar{n}\right)} & 0\\ 0 & 1+2\bar{n} & 0 & -2\mu \sqrt{\bar{n}\left(1+\bar{n}\right)}\\ 2\mu \sqrt{\bar{n}\left(1+\bar{n}\right)} & 0 & 1+2\bar{n} & 0\\ 0 & -2\mu \sqrt{\bar{n}\left(1+\bar{n}\right)} & 0 & 1+2\bar{n} \end{array}\right)$$

and the mean $d=(d_{A},d_{B})$. Take any Gaussian unitary operator $U_{S,m}$ such that $U_{S,m}\rho_{A}U_{S,m}^{†}=\rho_{A}$. Then, $SA_{0}S^{T}=A_{0}$ and $Sd_{A}+m=d_{A}$. As $S\Delta S^{T}=\Delta$, it is easily checked that there exists some $\theta \in [0,\frac{\pi }{2}]$ such that $S=S_{\theta }=\left(\begin{array}{cc} \cos\theta & \sin\theta \\ -\sin\theta & \cos\theta \end{array}\right)$. Therefore, the CM and the mean of $\left(U_{S,m}⊗I\right)\rho_{AB}\left(U_{S,m}^{†}⊗I\right)=\sigma_{AB}$ are respectively:

$$\begin{array}{c} \Gamma_{\theta }=\left(\begin{array}{cccc} a & 0 & c\cos\theta & d\sin\theta \\ 0 & a & -c\sin\theta & d\cos\theta \\ c\cos\theta & -c\sin\theta & b & 0\\ d\sin\theta & d\cos\theta & 0 & b \end{array}\right) \end{array}$$

and:

$$\left(S⊕I\right)\left(d_{A}⊕d_{B}\right)+m⊕0=\left(Sd_{A}+m\right)⊕d_{B}=d_{A}⊕d_{B}=\left(d_{A},d_{B}\right).$$

Note that, if the characteristic function of the Gaussian state is defined as $\chi_{\rho }\left(\alpha \right)=\exp\left[-\frac{1}{2}\lambda_{\alpha }^{T}J\tilde{\Gamma }J\lambda_{\alpha }+iJ{\tilde{d}}^{T}\lambda_{\alpha }\right]$, then, for any (1+1)-mode Gaussian states ρ,σ with CMs ${\tilde{V}}_{\rho },{\tilde{V}}_{\sigma }$ and means ${\tilde{d}}_{\rho },{\tilde{d}}_{\sigma }$, respectively, it was shown in [32,33] that:

(A2) $$F\left(\rho ,\sigma \right)=\frac{1}{\left(\sqrt{{\tilde{\Omega }}_{\rho \sigma }}+\sqrt{{\tilde{\Lambda }}_{\rho \sigma }}\right)-\sqrt{{\left(\sqrt{{\tilde{\Omega }}_{\rho \sigma }}+\sqrt{{\tilde{\Lambda }}_{\rho \sigma }}\right)}^{2}-{\tilde{\Upsilon }}_{\rho \sigma }}}\exp\left[-\frac{1}{2}\delta {\langle d\rangle }^{T}\det{\left[\left({\tilde{V}}_{\rho }+{\tilde{V}}_{\sigma }\right)\right]}^{-1}\delta \langle d\rangle \right],\;$$

where $\delta \langle d\rangle ={\tilde{d}}_{\rho }-{\tilde{d}}_{\sigma }$, ${\tilde{\Upsilon }}_{\rho \sigma }=\det\left({\tilde{V}}_{\rho }+{\tilde{V}}_{\sigma }\right)$, ${\tilde{\Omega }}_{\rho \sigma }=2^{4}\det\left[\left(J{\tilde{V}}_{\rho }\right)\left(J{\tilde{V}}_{\sigma }\right)-\frac{1}{4}I\right]$, and ${\tilde{\Lambda }}_{\rho \sigma }=2^{4}\det\left({\tilde{V}}_{\rho }+\frac{i}{2}J\right)\det\left({\tilde{V}}_{\sigma }+\frac{i}{2}J\right)$; while in this paper, we accept the characteristic function of the Gaussian state as $\chi_{\rho }\left(\alpha \right)=\exp\left[-\frac{1}{4}\lambda_{\alpha }^{T}J\Gamma J\lambda_{\alpha }+iJd^{T}\lambda_{\alpha }\right]$. Hence, for a Gaussian state ρ, one has $\Gamma =2\tilde{\Gamma }$. Especially, if the CM of the state is of the standard form, i.e., $\Gamma_{0}=\left(\begin{array}{cccc} a & 0 & c & 0\\ 0 & a & 0 & d\\ c & 0 & b & 0\\ 0 & d & 0 & b \end{array}\right)$ and: ${\tilde{\Gamma }}_{0}=\left(\begin{array}{cccc} \tilde{a} & 0 & \tilde{c} & 0\\ 0 & \tilde{a} & 0 & \tilde{d}\\ \tilde{c} & 0 & \tilde{b} & 0\\ 0 & \tilde{d} & 0 & \tilde{b} \end{array}\right)$, then $a=2\tilde{a}$, $b=2\tilde{b}$, $c=2\tilde{c}$, and $d=2\tilde{d}$. Therefore, for any (1+1)-mode Gaussian states ρ,σ with CMs $V_{\rho },V_{\sigma }$ and means $d_{\rho },d_{\sigma }$, respectively, by substituting ${\tilde{V}}_{\rho }=\frac{1}{2}V_{\rho }$ and ${\tilde{V}}_{\sigma }=\frac{1}{2}V_{\sigma }$ into Equation (A2), we get:

$$F\left(\rho ,\sigma \right)=\frac{1}{\left(\sqrt{\Omega_{\rho \sigma }}+\sqrt{\Lambda_{\rho \sigma }}\right)-\sqrt{{\left(\sqrt{\Omega_{\rho \sigma }}+\sqrt{\Lambda_{\rho \sigma }}\right)}^{2}-\Upsilon_{\rho \sigma }}}\exp\left[-\frac{1}{2}\delta {\langle d\rangle }^{T}\det{\left[\left(\frac{1}{2}V_{\rho }+\frac{1}{2}V_{\sigma }\right)\right]}^{-1}\delta \langle d\rangle \right],$$

where $\delta \langle d\rangle =d_{\rho }-d_{\sigma }$, $\Upsilon_{\rho \sigma }=\det\left(\frac{1}{2}V_{\rho }+\frac{1}{2}V_{\sigma }\right)$, $\Omega_{\rho \sigma }=2^{4}\det\left[\left(J\left(\frac{1}{2}V_{\rho }\right)\right)\left(J\left(\frac{1}{2}V_{\sigma }\right)\right)-\frac{1}{4}I\right]$, and $\Lambda_{\rho \sigma }=2^{4}\det\left(\frac{1}{2}V_{\rho }+\frac{i}{2}J\right)\det\left(\frac{1}{2}V_{\sigma }+\frac{i}{2}J\right)$. Using this formula, it is easily checked that:

$$N_{F}^{G}\left(\sigma_{AB}\right)=\max_{\theta \in [0,\frac{\pi }{2}]}\left(1-\frac{1}{\left(\sqrt{\Omega_{\theta }}+\sqrt{\Lambda }\right)-\sqrt{{\left(\sqrt{\Omega_{\theta }}+\sqrt{\Lambda }\right)}^{2}-\Upsilon_{\theta }}}\right),$$

where:

$$\Upsilon_{\theta }=\frac{1}{4}{\left[-2{\left(1+2\bar{n}\right)}^{2}+4\bar{n}\left(\bar{n}+1\right)\mu^{2}+4\bar{n}\left(\bar{n}+1\right)\mu^{2}\cos\theta \right]}^{2},$$

$$\Lambda =16{\bar{n}}^{4}{\left(1+\bar{n}\right)}^{4}{\left(-1+\mu^{2}\right)}^{4}$$

and:

$$\Omega_{\theta }={\left[1-2\bar{n}\left(-2+\mu^{2}\right)+8{\bar{n}}^{3}{\left(-1+\mu^{2}\right)}^{2}+4{\bar{n}}^{4}{\left(\mu^{2}-1\right)}^{2}+2{\bar{n}}^{2}\left(2\mu^{4}-5\mu^{2}+4\right)-2\bar{n}\left(1+\bar{n}\right)\mu^{2}\cos\theta \right]}^{2}.$$

Let:

$$\begin{array}{cc} f(\bar{n},\mu ,\theta )= & \sqrt{\Omega_{\theta }}+\sqrt{\Lambda }\\ = & 1-2\bar{n}\left(-2+\mu^{2}\right)+16{\bar{n}}^{3}{\left(-1+\mu^{2}\right)}^{2}+8{\bar{n}}^{4}{\left(-1+\mu^{2}\right)}^{2}\\ & +2{\bar{n}}^{2}\left(6-9\mu^{2}+4\mu^{4}\right)-2\bar{n}\left(1+\bar{n}\right)\mu^{2}\cos\theta \end{array}$$

and

$$\begin{array}{cc} g(\bar{n},\mu ,\theta )= & {\left(\sqrt{\Omega_{\theta }}+\sqrt{\Lambda }\right)}^{2}-\Upsilon_{\theta }\\ = & 16{\bar{n}}^{2}{\left(1+\bar{n}\right)}^{2}{\left(-1+\mu^{2}\right)}^{2}(1-2\bar{n}\left(-2+\mu^{2}\right)+8{\bar{n}}^{3}{\left(-1+\mu^{2}\right)}^{2}\\ & +4{\bar{n}}^{4}{\left(-1+\mu^{2}\right)}^{2}+2{\bar{n}}^{2}\left(4-5\mu^{2}+2\mu^{4}\right)-2\bar{n}\left(1+\bar{n}\right)\mu^{2}\cos\theta ). \end{array}$$

Then:

$$N_{F}^{G}\left(\sigma_{AB}\right)=\max_{\theta \in [0,\frac{\pi }{2}]}\left(1-\frac{1}{f\left(\bar{n},\mu ,\theta \right)-\sqrt{g(\bar{n},\mu ,\theta )}}\right)=\max_{\theta \in [0,\frac{\pi }{2}]}\left(1-\frac{1}{h(\bar{n},\mu ,\theta )}\right),$$

where

$$h\left(\bar{n},\mu ,\theta \right)=f\left(\bar{n},\mu ,\theta \right)-\sqrt{g(\bar{n},\mu ,\theta )}$$

with $\theta \in [0,\frac{\pi }{2}]$. We claim that $h(\bar{n},\mu ,\theta )$ is monotonically increasing in θ on the interval $\left[0,\frac{\pi }{2}\right]$. To see this, we need only to check $\frac{\partial h}{\partial \theta }>0$ on $\left(0,\frac{\pi }{2}\right)$. For $\theta \in (0,\frac{\pi }{2})$, a direct calculation gives that:

$$\frac{\partial h}{\partial \theta }=\frac{2\frac{\partial f}{\partial \theta }\sqrt{g}-\frac{\partial g}{\partial \theta }}{2\sqrt{g}}=\frac{\{4\bar{n}\left(1+\bar{n}\right)\mu^{2}\sqrt{g}-32{\bar{n}}^{3}{\left(1+\bar{n}\right)}^{3}\mu^{2}{\left(-1+\mu^{2}\right)}^{2}\}\sin\theta }{2\sqrt{g}}.$$

Since sinθ>0, it suffices to show that:

(A3) $$4\bar{n}\left(1+\bar{n}\right)\mu^{2}\sqrt{g}-32{\bar{n}}^{3}{\left(1+\bar{n}\right)}^{3}\mu^{2}{\left(-1+\mu^{2}\right)}^{2}>0.\;$$

Note that:

$$\begin{array}{cc} & 4\bar{n}\left(1+\bar{n}\right)\mu^{2}\sqrt{g}-32{\bar{n}}^{3}{\left(1+\bar{n}\right)}^{3}\mu^{2}{\left(-1+\mu^{2}\right)}^{2}>0\\ \Leftrightarrow & \sqrt{g}>8{\bar{n}}^{2}{\left(1+\bar{n}\right)}^{2}{\left(-1+\mu^{2}\right)}^{2}\\ \Leftrightarrow & g>64{\bar{n}}^{4}{\left(1+\bar{n}\right)}^{4}{\left(-1+\mu^{2}\right)}^{4}\\ \Leftrightarrow & \cos\theta <\frac{1-2\bar{n}\left(-2+\mu^{2}\right)-2{\bar{n}}^{2}\left(-2+\mu^{2}\right)}{2\bar{n}\left(1+\bar{n}\right)\mu^{2}}. \end{array}$$

Let:

$$\psi \left(\bar{n},\mu \right)=\frac{1-2\bar{n}\left(-2+\mu^{2}\right)-2{\bar{n}}^{2}\left(-2+\mu^{2}\right)}{2\bar{n}\left(1+\bar{n}\right)\mu^{2}}.$$

It is easily checked that $\psi (\bar{n},\mu )>1$ holds for any $\bar{n}\geq 0$ and 0≤μ≤1. Consequently, $\cos\theta <\psi (\bar{n},\mu )$, which implies that Equation (A3) is true, as desired. Therefore, $\frac{\partial h}{\partial \theta }>0$ and $h(\bar{n},\mu ,\theta )$ is a monotonically-increasing function in θ. It follows that:

$$\begin{array}{cc} N_{F}^{G}\left(\rho_{AB}\right)=N_{F}^{G}\left(\sigma_{AB}\right) & =\max_{\theta \in [0,\frac{\pi }{2}]}\left(1-\frac{1}{\left(\sqrt{\Omega_{\theta }}+\sqrt{\Lambda }\right)-\sqrt{{\left(\sqrt{\Omega_{\theta }}+\sqrt{\Lambda }\right)}^{2}-\Upsilon_{\theta }}}\right)\\ & =\max_{\theta \in [0,\frac{\pi }{2}]}\left(1-\frac{1}{f\left(\bar{n},\mu ,\theta \right)-\sqrt{g(\bar{n},\mu ,\theta )}}\right)\\ & =1-\frac{1}{f\left(\bar{n},\mu ,\frac{\pi }{2}\right)-\sqrt{g(\bar{n},\mu ,\frac{\pi }{2})}}\\ & =1-\frac{1}{\left(\sqrt{\Omega_{\frac{\pi }{2}}}+\sqrt{\Lambda }\right)-\sqrt{{\left(\sqrt{\Omega_{\frac{\pi }{2}}}+\sqrt{\Lambda }\right)}^{2}-\Upsilon_{\frac{\pi }{2}}}}. \end{array}$$

The proof is completed by taking $\Omega =\Omega_{\frac{\pi }{2}}$ and $\Upsilon =\Upsilon_{\frac{\pi }{2}}$. Finally, it is easily checked that:

$$\lim_{\bar{n}\to \infty }\left[\left(\sqrt{\Omega }+\sqrt{\Lambda }\right)-\sqrt{{\left(\sqrt{\Omega }+\sqrt{\Lambda }\right)}^{2}-\Upsilon }\right]=\infty$$

and hence, $\lim_{\bar{n}\to \infty }N_{F}^{G}\left(\rho_{AB}\left(\bar{n},\mu \right)\right)=1$. ☐
